# NAP1L1 promotes proliferation and chemoresistance in glioma by inducing CCND1/CDK4/CDK6 expression through its interaction with HDGF and activation of c-Jun

**DOI:** 10.18632/aging.203805

**Published:** 2021-12-27

**Authors:** Zigui Chen, Yingying Xie, Hongcheng Luo, Ye Song, Tianshi Que, Rentong Hu, Huatuo Huang, Kunxiang Luo, Chuanyu Li, Chengjian Qin, Chuanhua Zheng, Weiyi Fang, Longyang Liu, Hao Long, Qisheng Luo

**Affiliations:** 1 Neuroscience Center, Cancer Center, Integrated Hospital of Traditional Chinese Medicine, Southern Medical University, Guangzhou 510315, China; 2 Southern Medical University, Guangzhou, Guangdong 510000, China; 3 Department of Laboratory Medicine, Affiliated Hospital of Youjiang Medical University for Nationalities, Guangxi, Baise 53300, China; 4 Department of Neurosurgery, Nanfang Hospital, Southern Medical University, Guangzhou, Guangdong 510515, China; 5 Department of Neurosurgery, Affiliated Hospital of Youjiang Medical University for Nationalities, Guangxi, Baise 53300, China

**Keywords:** NAP1L1, HDGF, glioma, proliferation, chemoresistance

## Abstract

The prognosis of glioma is poor as its pathogenesis and mechanisms underlying cisplatin chemoresistance remain unclear. Nucleosome assembly protein 1 like 1 (NAP1L1) is regarded as a hallmark of malignant tumors. However, the role of NAP1L1 in glioma remains unknown. In this study, we aimed to investigate the molecular functions of NAP1L1 in glioma and its involvement in cisplatin chemoresistance, if any. NAP1L1 was found to be upregulated in samples from The Cancer Genome Atlas (TCGA) database. Immunohistochemistry indicated that NAP1L1 and hepatoma-derived growth factor (HDGF) were enhanced in glioma as compared to the para-tumor tissues. High expressions of NAP1L1 and HDGF were positively correlated with the WHO grade, KPS, Ki-67 index, and recurrence. Moreover, NAP1L1 expression was also positively correlated with the HDGF expression in glioma tissues. Functional studies suggested that knocking down NAP1L1 could significantly inhibit glioma cell proliferation both *in vitro* and *in vivo*, as well as enhance the sensitivity of glioma cells to cisplatin (cDDP) *in vitro*. Mechanistically, NAP1L1 could interact with HDGF at the protein level and they co-localize in the cytoplasm. HDGF knockdown in NAP1L1-overexpressing glioma cells significantly inhibited cell proliferation. Furthermore, HDGF could interact with c-Jun, an oncogenic transcription factor, which eventually induced the expressions of cell cycle promoters, CCND1/CDK4/CDK6. This finding suggested that NAP1L1 could interact with HDGF, and the latter recruited c-Jun, a key oncogenic transcription factor, that further induced CCND1/CDK4/CDK6 expression, thereby promoting proliferation and chemoresistance in glioma cells. High expression of NAP1L1 in glioma tissues indicated shorter overall survival in glioma patients.

## INTRODUCTION

Glioma is the most commonly diagnosed intracranial malignancy. Currently, extended surgical resection combined with radiotherapy or chemotherapy is the main treatment strategies [1]. Due to the heterogeneity of tumor-related genes and tumor susceptibility, the prognosis of patients with glioma remains poor, thereby making it a big problem among patients with intracranial tumors [2, 3]. Despite the novel therapeutic approaches targeted for glioma treatment, the death rate remains consistently high and their 5-year prognosis remains low. The poor effects of therapy may be attributed to the high proliferation rates of glioma cells and their resistance to chemotherapy. Therefore, it is important to identify novel molecular markers and develop corresponding treatment strategies to improve the prognosis of patients with glioma.

Nucleosome Assembly Protein 1 Like 1, NAP1L1, also known as NRP or NAP1, is located at 12q21.2 and belongs to the family of nucleosome assembly proteins. NAP1L1 is present in most human tissues and cell lines, however, upregulated levels are often detected in rapidly proliferating cells [4]. Several studies report the high NAP1L1 expression in tumors [5–8], which points to its potential role in these malignancies. Although as early as the 1990s, Japanese scientists showed that NAP1 could accelerate the assembly of nucleosomes and participate in cell DNA replication and chromatin regulation [9], it is only in recent years that studies have shown its close association with cell proliferation and other functions in stem cells. Nevertheless, the biological role as well as the molecular mechanism of NAP1L1 underlying glioma onset and progression remains unknown. In our previous study, NAP1L1 was found to be significantly upregulated in glioma as compared to the para-tumor tissues and high NAP1L1 expression was associated with poor survival in patients with glioma. NAP1L1 is an oncogene, and its knockdown inhibits glioma cell proliferation. But the comprehensive molecular mechanisms of NAP1L1 underlying glioma onset and progression remain unclear. These results would provide new insights to illustrate the molecular mechanism of glioma development.

In this study, we investigated specific molecular functions and roles of NAP1L1 in glioma cells, thereby providing evidence that NAP1L1 could promote cell proliferation and chemoresistance in glioma cells by interacting with HDGF and subsequently, activating c-Jun to induce CCND1/CDK4/CDK6 expressions. Our findings implicated NAP1L1 as a potential diagnostic biomarker and therapy target for glioma.

## RESULTS

### NAP1L1 is strongly expressed in glioma tissues and correlates with low prognosis

**Figure 1:**
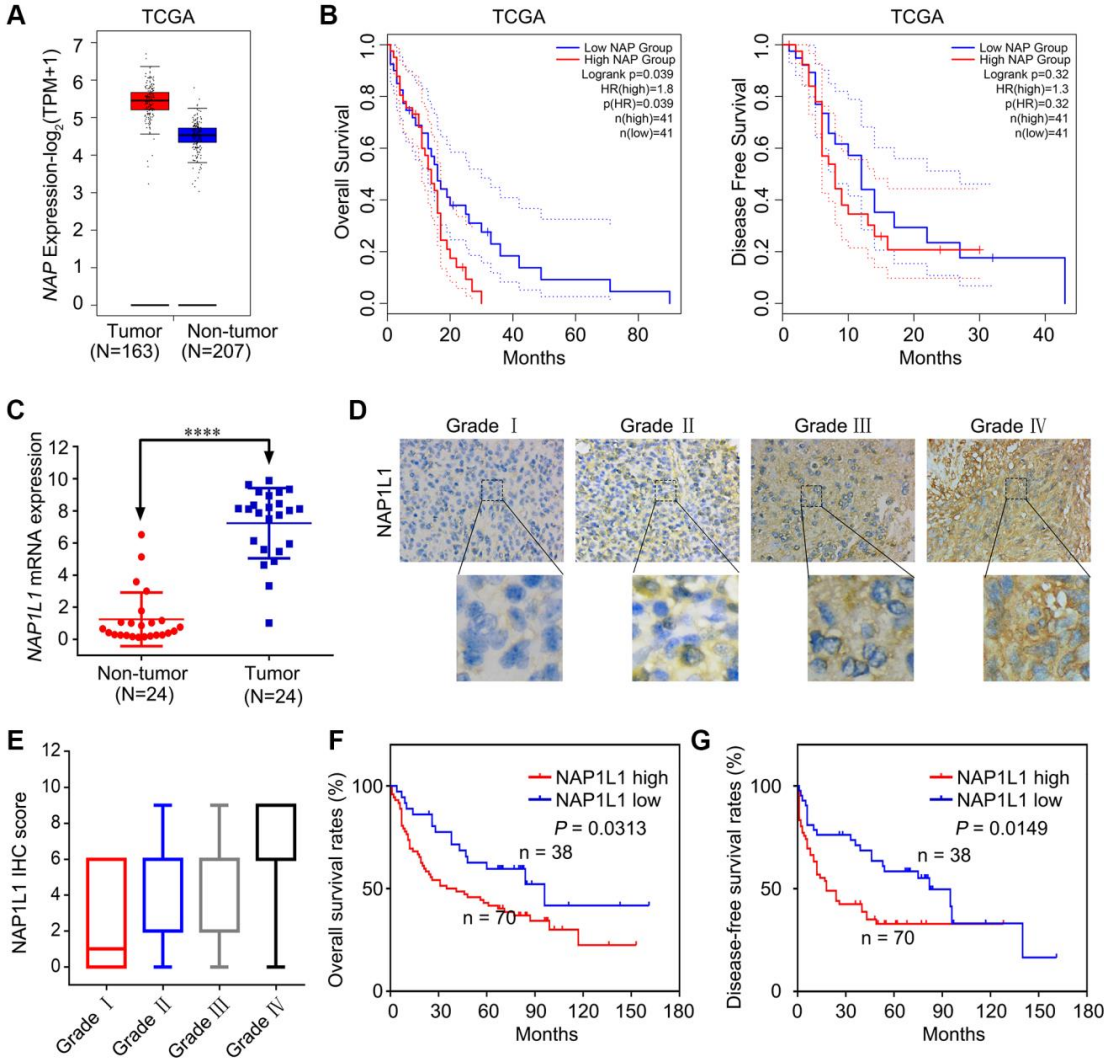
**NAP1L1 is highly expressed in glioma and correlates with poor prognosis.** (**A**) NAP1L1 mRNA expression in glioma tissues and para-tumor tissues among the glioma patients obtained from the TCGA database. (**B**) Kaplan-Meier survival analysis for overall survival based on the NAP1L1 expression data. (**C**) RT-qPCR analysis of NAP1L1 mRNA expression in 24 glioma tissues and 24 para-tumor tissues. (**D**, **E**) Representative images of NAP1L1 staining (**D**) and IHC score (**E**) of in (grade I–IV) glioma tissues (scale bar: 50 μm). (**F**, **G**) Kaplan-Meier survival analysis for overall survival (**F**) and Disease Free survival (**G**) in TMA showing NAP1L1 expression. Data are presented as the mean ± SD for three independent experiments. ^*^P < 0.05, ^**^P < 0.01, ^***^P < 0.001.

Several previous studies show that NAP1L1 expression is strictly correlated with malignant neoplastic progression in several tumor types [5–8]. Based on the analysis of samples from the TCGA database, we found increased expression of NAP1L1 at the mRNA level (Figure 1A). Analysis of OS and disease free survival (DFS) showed that overexpression of NAP1L1 was a negative factor that decreased the overall survival duration in glioma patients (Figure 1B). The RT-qPCR analysis also confirmed that NAP1L1 mRNA expression was significantly enhanced in 24 gliomas tissues as compared to the 24 corresponding para-tumor tissues (*P* < 0.001) (Figure 1C). A tissue microarray (TMA) with 108 glioma tissue samples and 24 para-tumor tissues was used to assess the expression level of NAP1L1 (Figure 1D, 1E). The survival analysis demonstrated that overexpression of NAP1L1 was an adverse factor that decreased the survival duration of glioma patients (Figure 1F, 1G). As shown in Table 1, high expression of NAP1L1 in glioma tissues was significantly related to WHO grades I~IV, KPS <80, Ki-67 index ≥20%, and recurrence. Furthermore, univariate and multivariate cox regression analyses showed that NAP1L1 was a disadvantage predictive factor, which indicated poor survival in glioma patients (Tables 2 and 3).

**Table 1:** The correlation of NAP1L1 or HDGF protein expression with clinicopathological parameters in gliomas.

**Characteristics**	***N***	**NAP1L1**		***P* value**	**HDGF**		***P* value**
		**Low**	**High**		**Low**	**High**	
Age (years)							
<55	86	29	57	0.866	36	50	0.936
≥55	22	7	15		9	13	
Gender							
Male	59	21	38	0.585	27	32	0.343
Female	49	15	34		18	31	
WHO grade							
I	7	6	1	**0.013**	6	1	**0.026**
II	43	15	28		21	22	
III	18	6	12		6	12	
IV	40	9	31		12	28	
Ki-67 index							
≥20%	60	12	48	**0.001**	19	41	**0.018**
<20%	48	24	24		26	22	
KPS							
≥80	84	32	52	**0.050**	40	44	**0.019**
<80	24	4	20		5	19	
Vital status							
Alive	45	21	24	**0.013**	26	19	**0.004**
Dead	63	15	48		19	44	
Recurrence							
No	23	16	7	**<0.001**	16	7	**0.002**
Yes	85	20	65		29	56	

**Table 2:** Univariate analysis of factors associated with OS and DFS in 108 glioma patients.

**Factors**	**OS**		***P*-value**	**DFS**		***P*-value**
	**HR**	**95% CI**		**HR**	**95% CI**	
Age	0.536	0.302–0.951	**0.033**	0.665	0.393–1.126	0.129
Gender	1.232	0.750–2.022	0.410	1.042	0.676–1.604	0.853
WHO grade	1.868	1.425–2.448	**<0.001**	1.438	1.162–1.780	**0.001**
Ki-67 index	0.336	0.193–0.585	**<0.001**	0.400	0.252–0.633	**<0.001**
KPS score	4.257	2.449–7.398	**<0.001**	3.468	2.060–5.841	**<0.001**
HDGF	2.213	1.288–3.802	**0.004**	2.174	1.371–3.446	**0.001**
NAP1L1	2.081	1.164–3.723	**0.013**	2.399	1.443–3.989	**0.001**

**Table 3:** Multivariate analysis of factors associated with OS and DFS in 108 glioma patients.

**Factors**	**OS**		***P*-value**	**DFS**		***P*-value**
	**HR**	**95% CI**		**HR**	**95% CI**	
**Age**	0.574	0.317–1.040	0.067			
**WHO grade**	1.445	1.040–2.006	**0.028**	1.096	0.839–1.431	0.503
**Ki-67 index**	0.688	0.347–1.366	0.286	0.671	0.353–1.081	0.091
**KPS score**	2.393	1.306–4.386	**0.005**	2.341	1.334–4.110	**0.003**
**NAP1L1**	1.372	0.740–1.040	0.315	1.768	1.035–3.020	**0.037**

### Suppressing NAP1L1 inhibits glioma cell proliferation and chemoresistance

**Figure 2:**
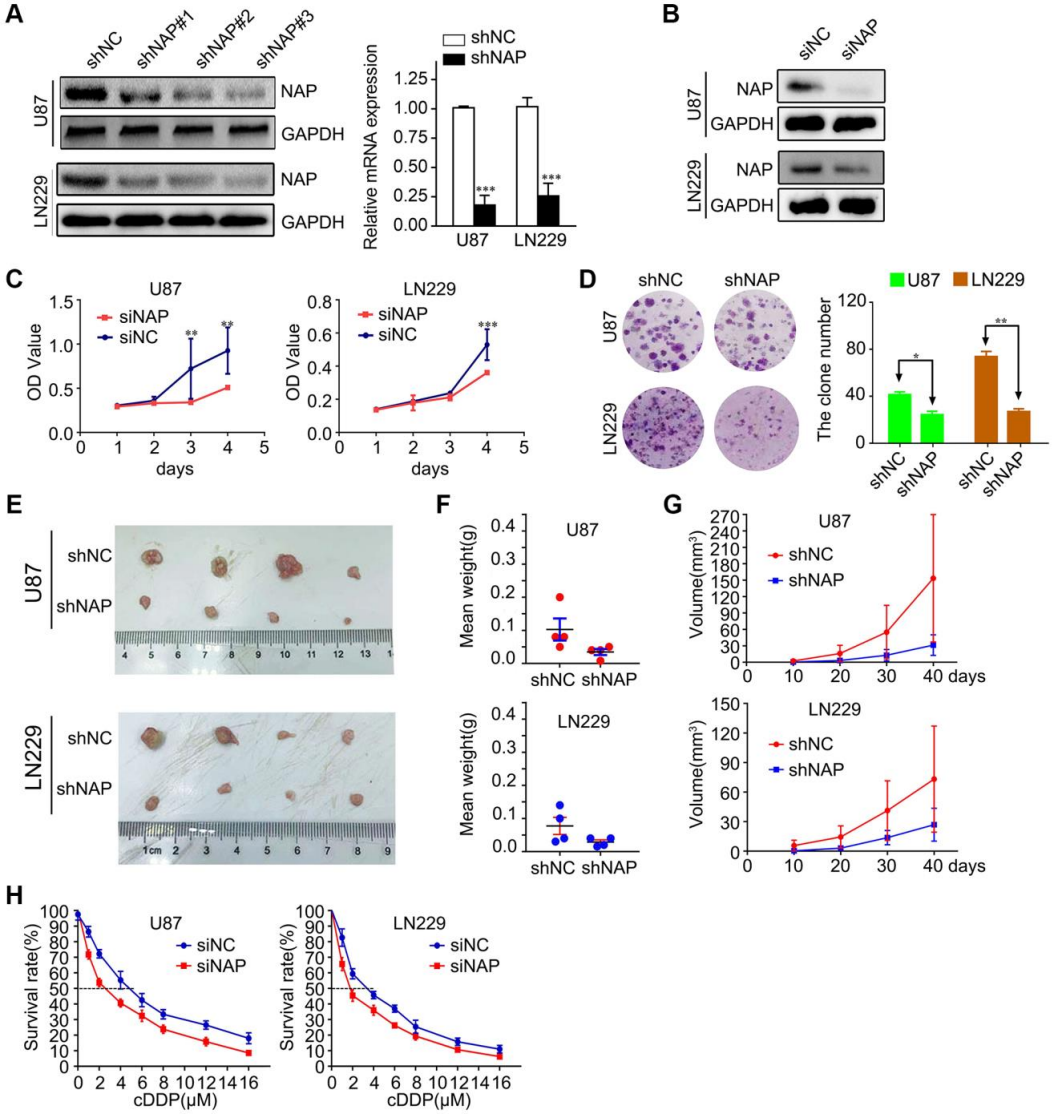
**Suppressing NAP1L1 inhibits glioma cell proliferation and chemoresistance.** (**A**) NAP protein level was measured by western blot in U87 and LN229 cells transfected with shNC or shNAP. GAPDH was used as a loading control. With efficient knockdowns from shNAP was separately detected by RT-PCR assays. (**B**) NAP protein level was measured by western blot in U87 and LN229 cells transfected with siNC or siNAP. GAPDH was used as a loading control. MTT assay (**C**), clone formation assay (**D**) after NAP1L1 knockdown. Gross morphology of tumors (**E**) and tumor weight statistics (**F**) from the indicated groups (*n* = 4 per group). (**G**) Tumor volume statistics for each mouse group (*n* = 4 per group). (**H**) Dose-response curves of U87 and LN229 treated with shNAP and shNC respectively following treatment with DDP for 48 h. Data are presented as the mean ± SD for three independent experiments. ^*^P < 0.05, ^**^P < 0.01, ^***^P < 0.001.

To study the potential role of NAP1L1 in the progression of glioma, we transfected LN229 and U87 cells with lentiviral constructs expressing short hairpin RNA targeting NAP1L1 and the corresponding negative control (shNC) (Supplementary Figure 1A). The protein level expression of NAP1L1 was assessed by western blotting (Figure 2A). After confirming efficient knockdown using shNAP1L1 in the LN229 cell line, the same fragment in the U87 cell line was separately detected by RT-PCR assay and compared with the shNC group (Figure 2A). We also assessed the interference effects from small interfering RNAs (siRNAs) that were used to knock down the endogenous NAP1L1 as compared to the negative control (si-NC) group (Figure 2B).

Next, we investigated the effect of attenuated expression of NAP1L1 on the growth of glioma cells *in vitro*. The MTT assay demonstrated that NAP1L1 knockdown could significantly decrease the glioma cells proliferation as compared to that of the control (si-NC) group (Figure 2C). In addition, clone formation assays further confirmed that shNAP1L1-mediated knockdown in glioma cells significantly inhibited cell proliferation (Figure 2D). Moreover, a model of subcutaneous xenograft was established in nude mice with shNAP1L1-transfected glioma cells. We found that NAP1L1 knockdown, that is, in mice that carried the shNAP1L1 glioma cells, resulted in the formation of smaller tumors as compared to those in the control (sh-NC) group (Figure 2E–2G).

Interestingly, glioma cells with stably silenced NAP1L1 showed significantly enhanced sensitivity to cisplatin (DDP) treatment. Glioma cells were processed with various concentrations of DDP after 48 h and the cell growth inhibition rates were calculated after NAP1L1 silencing. The IC50 value of DDP was 3.38 μM in the parental LN229 cells but decreased significantly to 1.56 μM in NAP1L1-silenced LN229 cells (*P* < 0.05); a similar IC50 reduction from 4.84 μM to 2.58 μM was observed in the U87 cells upon silencing (Figure 2H). Moreover, silenced NAP1L1 also increased sensitivity to temozolomide and the results were showed in Supplementary Figure 1B.

### NAP1L1 controls the expression of genes associated with cell cycle and apoptosis through the CCND1/CDK4/CDK6 signaling pathway in glioma

**Figure 3:**
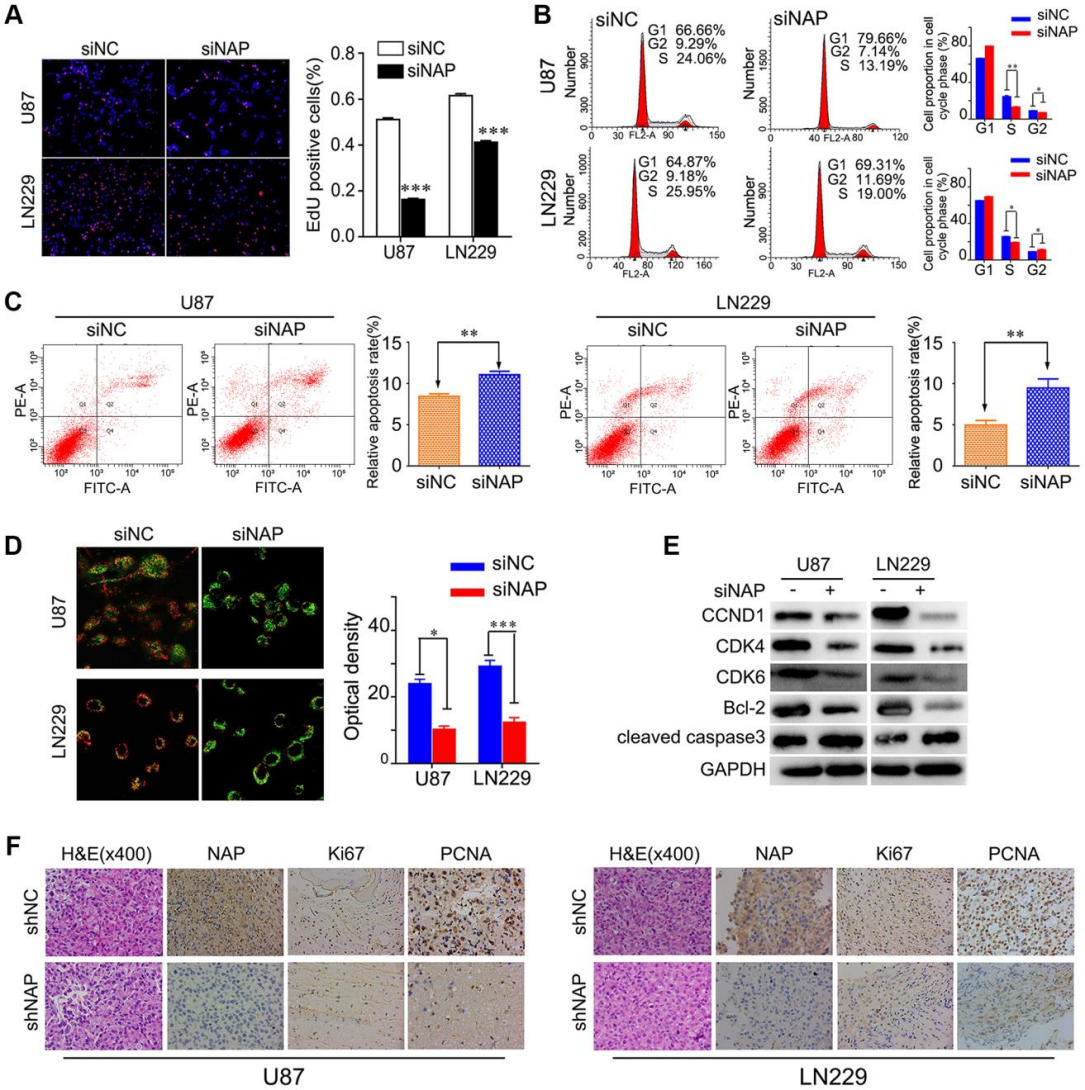
**NAP1L1 controls the expression of cell cycle and apoptosis associated genes via the CCND1/CDK4/CDK6 signaling pathways in glioma.** EdU incorporation assay (**A**), Cell cycle analysis (**B**), Cell apoptosis assay (**C**) Mitochondrial membrane potential assay (**D**) in U87 and LN229 cells transfected with control siRNA (siNC) or NAP1L1 siRNA (siNAP). (**E**) Western blotting analysis of the protein levels of CCND1, CDK4, CDK6, Bcl-2 and cleaved caspase3 after transfecting siNC or siNAP into U87 and LN229 cells. (**F**) The H&E of the nude mice tumor tissues. NAP1L1, Ki67, and PCNA was evaluated by immunohistochemical staining. Compared with shNAP cells, the shNC cell tumor tissues were high expression. ^*^P < 0.05, ^**^P < 0.01, ^***^P < 0.001.

To further study the mechanism underlying NAP1L1-silencing mediated reduction in cell proliferation, we performed EdU analysis. The findings showed that NAP1L1 knockdown significantly inhibited the proliferation of glioma cells in comparison with that in the control group (Figure 3A). Cell cycle analysis showed that the suppression of NAP1L1 could significantly reduce the cell cycle progression from G1 to S phase (Figure 3B). The cell apoptosis assay and mitochondrial membrane potential assay indicated that siNAP1L1 dramatically induced apoptosis in glioma cells (Figure 3C, 3D). Interestingly, glioma cells with stably silenced NAP1L1 also significantly inhibited the proliferation. The results were showed in Supplementary Figure 2A–2D. In addition, we investigated the protein levels of genes associated with cell cycle and apoptosis in the U251 and U87 glioma cells with stable suppression of NAP1L1. Western blotting demonstrated that siNAP1L1 inhibited the activation of oncogenic cell cycle regulators, such as CDK4, CDK6, and CCND1 (Figure 3E). Furthermore, a potent anti-apoptotic regulatory factor of Bcl-2 significantly decreased in the U251 and U87 cells after transfection with siNAP1L1. As well, the protein level of cleaved caspase-3 was upregulated in both the cell lines with stably suppressed NAP1L1 (Figure 3E). Moreover, the mice injected with shNAP1L1-U87 and shNAP1L1-LN229 cells exhibited lower expressions of Ki67, proliferating cell nuclear antigen (PCNA) and NAP1L1 in tumor tissues relative to the control mice (Figure 3F). These data demonstrated that NAP1L1 could dramatically promote cell proliferation through the CDK4/CDK6/CCND1 signaling axis in glioma.

### NAP1L1 interacts with HDGF and HDGF knockdown reverses the proliferative effect in glioma cells overexpressing NAP1L1

**Figure 4:**
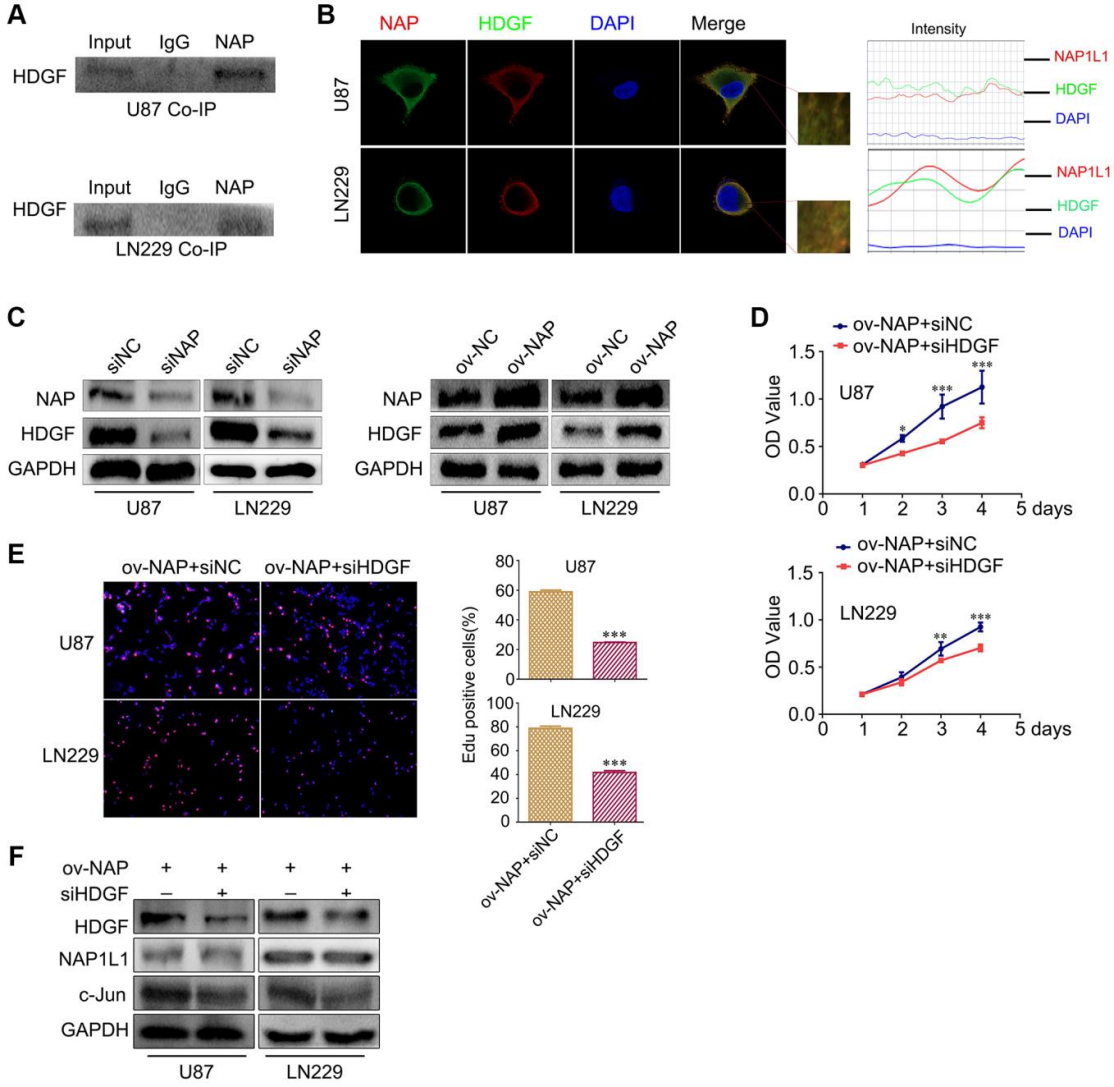
**NAP1L1 interacts with HDGF and HDGF knockdown reverses the effect of overexpressed NAP1L1 on proliferation of glioma cells.** (**A**) Co-IP experiments detected the interaction of endogenous NAP1L1 and HDGF in U87 and LN229 cells. (**B**) Representative immunofluorescence staining and intensity of NAP1L1 and HDGF protein in U87 and LN229 cells. Scale bar, 5 μm. (**C**) HDGF level in U87 and LN229 cells transfected with siNAP1L1 or NAP1L1-overexpressing plasmid. MTT assay (**D**) and EdU incorporation assay (**E**) in glioma cells transfected with control or HDGF siRNA. (**F**) Western blotting analysis of the protein levels of HDGF, NAP1L1 and c-Jun after transfection of siHDGF into glioma cells. GAPDH served as the internal control. Data are presented as the mean ± SD for three independent experiments. ^*^P < 0.05, ^**^P < 0.01, ^***^P < 0.001.

In our preliminary work, using mass spectrometry, we predicted a direct interaction between NAP1L1 and HDGF proteins, and this was confirmed in endometrial carcinoma (unpublished data). Thus, coimmunoprecipitation (co-IP) was performed to examine the relevant binding partners of NAP1L1. Endogenous co-IP assay indicated an interaction between HDGF and NAP1L1 in the U251 and U87 cells (Figure 4A). Immunofluorescence assay confirmed their colocalization in the cytoplasm of glioma cells (Figure 4B). In addition, NAP1L1 overexpression could upregulate HDGF expression in glioma cells, while NAP1L1 knockdown decreased the protein level expression of HDGF (Figure 4C) (Supplementary Figure 2E). To further evaluate the relationship between HDGF and NAP1L1, we performed MTT and EdU assays using NAP1L1-overexpressing glioma cells upon HDGF knockdown (Figure 4D, 4E). The results indicated that HDGF knockdown could significantly reverse NAP1L1 overexpression-mediated enhanced cell proliferation; the findings from western blotting further confirmed this result (Figure 4F). These results demonstrated that NAP1L1 interacted with HDGF, and further, induced cell proliferation in glioma.

### HDGF interacts with c-Jun and c-Jun overexpression reverses the proliferative effect upon HDGF knockdown in glioma cells

**Figure 5:**
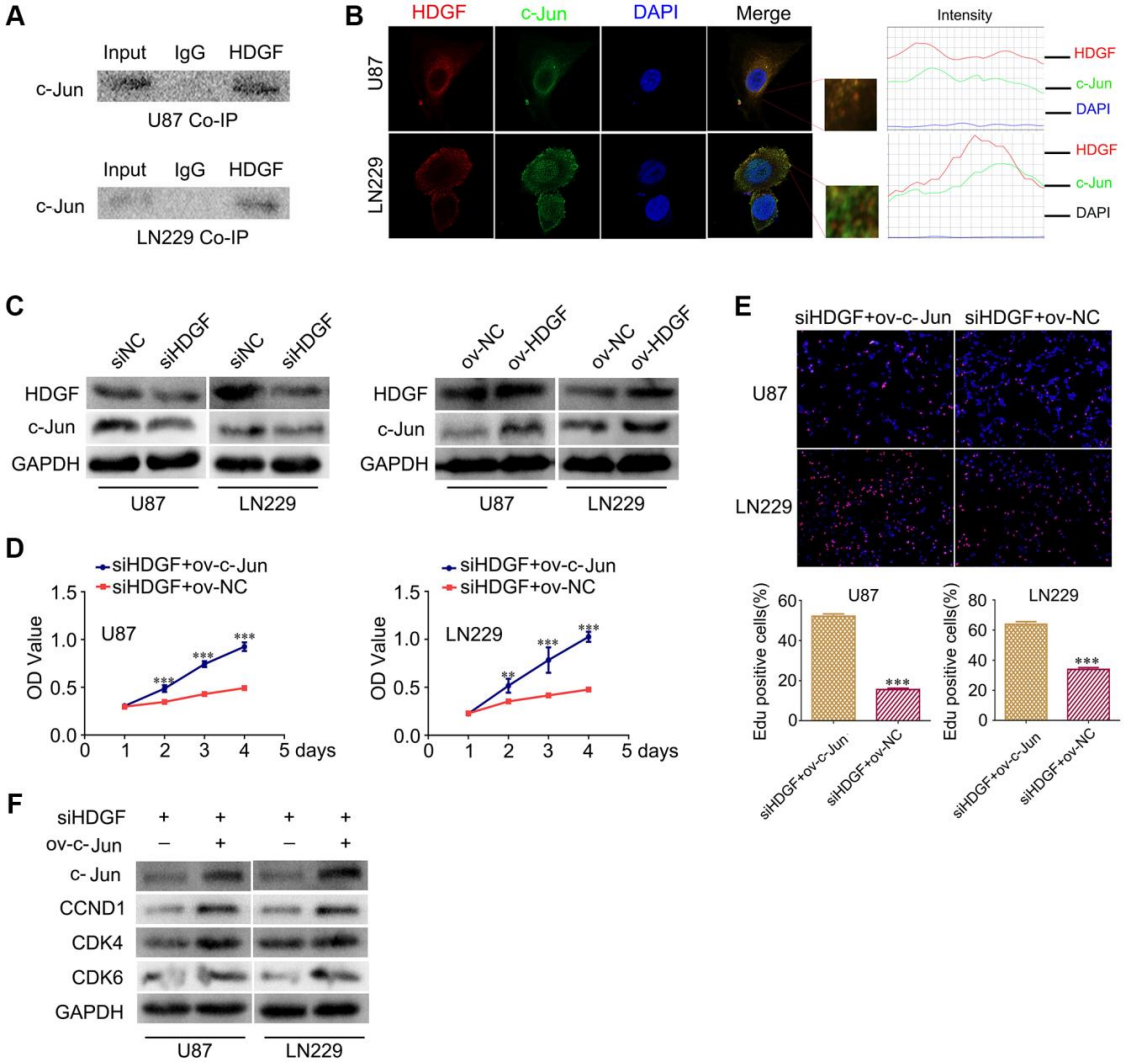
**HDGF interacts with c-Jun and c-Jun overexpression reverses the effect of HDGF knockdown on proliferation of glioma cells.** (**A**) Co-IP experiments detected the interaction of endogenous HDGF and c-Jun in U87 and LN229 cells. (**B**) Representative immunofluorescence staining and intensity of HDGF and c-Jun protein in U87 and LN229 cells. Scale bar, 5 μm. (**C**) c-Jun expression in U87 and LN229 cells transfected with siHDGF or HDGF-overexpressing plasmid. The MTT assay (**D**) and EdU incorporation assay (**E**) in glioma cells after transfecting c-Jun-overexpressing plasmid. (**F**) Western blotting for c-Jun, CCND1, CDK4 and CDK6 in glioma cells after transfected with c-Jun-overexpressing plasmid. GAPDH served as the internal control. Data are presented as the mean ± SD for three independent experiments. ^*^P < 0.05, ^**^P < 0.01, ^***^P < 0.001.

To further clarify the mechanism of NAP1L1-mediated enhanced cell proliferation through HDGF in glioma cells, The BIOGRID database was used to identify the proteins that could interact with HDGF. We predicted that HDGF and c-Jun would interact directly. Thus, co-IP was completed to examine the relevant binding partners of HDGF. The endogenous co-IP assay verified the interaction between HDGF and c-Jun in the U251 and U87 cells (Figure 5A). Immunofluorescence assay showed a strong co-localization signal mainly in the cytoplasm; minor nuclear distribution was also observed (Figure 5B). Moreover, HDGF overexpression could upregulate c-Jun expression in glioma cells, while HDGF knockdown attenuated c-Jun expression at the protein level (Figure 5C) (Supplementary Figure 2F). To further evaluate the relationship between HDGF and c-Jun, MTT assay and EdU assays were completed to examine the reversal of the effect of c-Jun on HDGF knockdown in glioma cell proliferation (Figure 5D, 5E). Western blotting illustrated that c-Jun/CCND1/CDK4/CDK6 signaling was dramatically enhanced (Figure 5F). Taken together, these results demonstrated that c-Jun enhanced upon HDGF overexpression could promote glioma cell proliferation by inducing the activation of the CCND1/CDK4/CDK6 signaling axis.

### Correlation of NAP1L1 and HDGF expression with overall survival in patients with glioma

**Figure 6:**
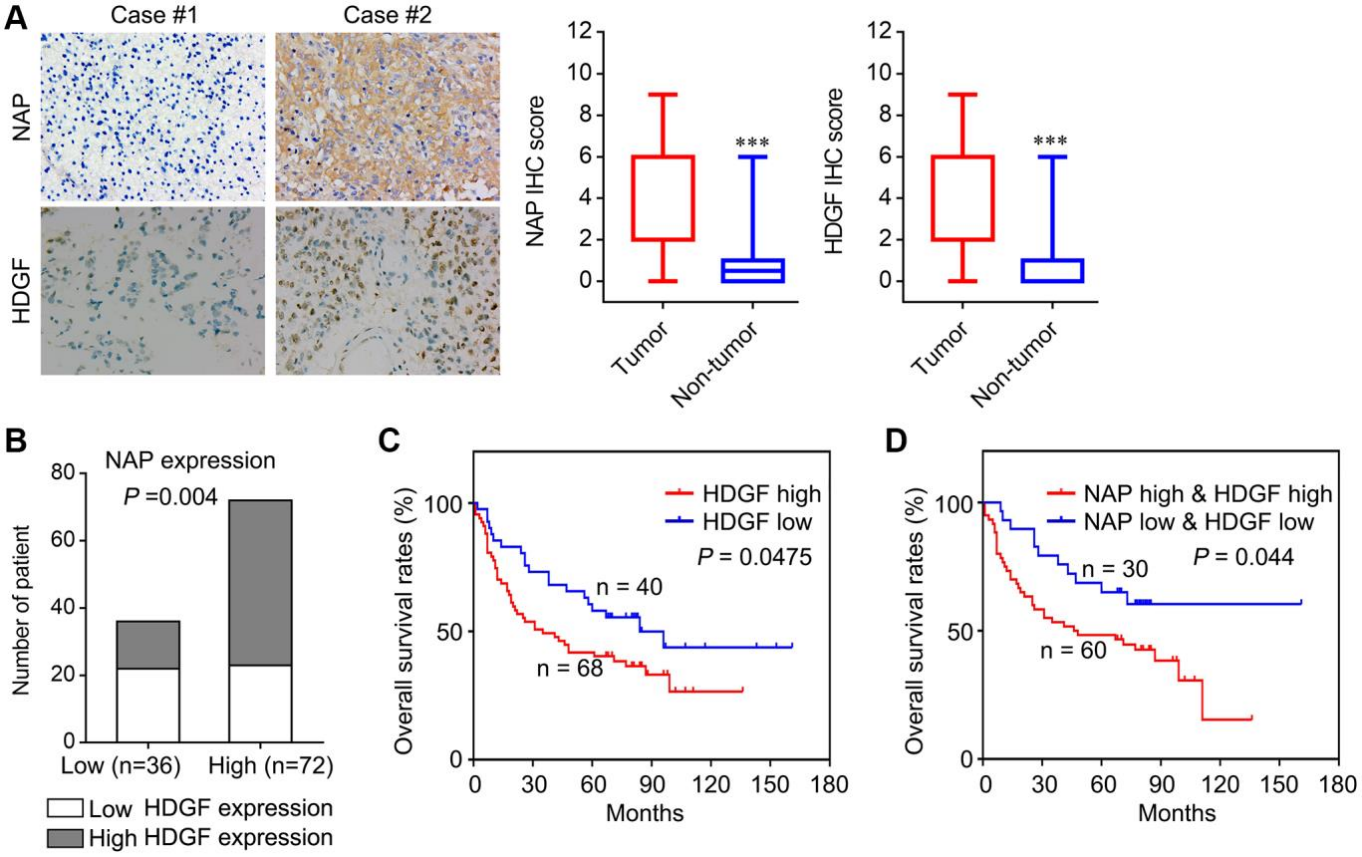
**Correlation analysis of NAP1L1 and HDGF with overall survival of patients with glioma.** (**A**) Representative immunohistochemistry images and IHC score of expression of NAP1L1 and HDGF in glioma tissues, respectively. Original magnification 400×. (**B**) Correlation analysis of NAP1L1 and HDGF with overall survival of patients with glioma. Prognostic significance assessed by Kaplan-Meier survival estimates. Comparison of the overall survival by HDGF (**C**), respectively. (**D**) Kaplan-Meier analysis of overall survival in patients with variable expression of NAP1L1 and HDGF. ^*^P < 0.05, ^**^P < 0.01, ^***^P < 0.001.

To determine the role of NAP1L1 and HDGF in glioma, we analysed the protein level expressions of NAP1L1 and HDGF by immunostaining in 108 glioma tissues and 24 para-tumor tissues (Figure 6A). Immunohistochemical (IHC) staining for the expressions of NAP1L1 and HDGF showed that NAP1L1 positive signals were mostly localized to the cytoplasm of glioma cells, while those of HDGF was mainly in the cytoplasm and nuclei (Figure 6A). In addition, IHC analysis demonstrated that tumors having high expression of NAP1L1 also had significantly higher expression of HDGF as compared to those with low expression of NAP1L1 (Figure 6B). Next, the prognostic implications of NAP1L1 and HDGF in gliomas were assessed. Kaplan-Meier survival analysis illustrated that patients with high expression levels of HDGF had dramatically shortened OS times (Figure 6C) compared with patients with low level of HDGF expression. Interestingly, patients with both high HDGF and NAP1L1 expressions had the shortest survival duration (Figure 6D).

To verify the functions of NAP1L1 and HDGF, we analyzed the correlation of NAP1L1 and HDGF expressions with widely recognized clinicopathological parameters in glioma specimens. As shown in Table 1, high HDGF expressions in gliomas were significantly associated with WHO grades II~IV, KPS <80, Ki-67 index ≥20%, and recurrence. Furthermore, univariate analysis showed that the WHO grade, KPS, Ki-67 index, recurrence, NAP1L1 and HDGF expressions were unfavorable predictors for overall survival (Table 2). Correlation analysis indicated a significantly positive correlation between NAP1L1 and HDGF expressions in glioma tissues (Table 4, r = 0.279, *P* = 0.003).

**Table 4:** Correlation between NAP1L1 and HDGF expression in glioma tissues.

		**NAP1L1 expression**		***r-*Value**	***P-*Value**
		**Low**	**High**		
**HDGF expression**	**Low**	22	23	0.279	0.003
	**High**	14	49		

## DISCUSSION

Despite huge progress in the development of diagnostic and therapeutic methods, including radiation treatment and chemotherapy, glioma remains one of the most lethal cancers in humans [10, 11]. The average survival period of patients with glioma is less than two years and the five-year survival rate is less than 3%, the lowest among all cancers [12]. Thus, it is important to develop novel diagnostic methods and effective treatment strategies. NAP1L1 is the human analog of the yeast NAP-I protein, a histone-binding factor involved in the maintenance of cumulative nucleosome formation [13]. In humans, NAP1L1 belongs to a family of proteins involved in nucleosome assembly and transcriptional regulation [14]. Both mRNA and protein levels of NAP1L1 increase rapidly in conjunction with cell proliferation in a T-lymphoid cell model [15]. NAP1L1 is upregulated in small-intestinal neuroendocrine cell-derived neoplastic tissues [16], colon cancer [17], and malignant adenocarcinoma as compared to normal mucosa [18]. NAP1L1 is involved in the ovarian cancer cell response to cytotoxic gold compounds [19]. In the last two decades, extensive studies show that NAP1L1 is an important regulator, critical for various biological processes in multiple cancer types [5, 8]. However, its role in human glioma remains unknown.

To address the problem, first, we analysed the mRNA level of NAP1L1 in the TCGA database samples. The data demonstrated that at the mRNA level, the expression of NAP1L1 was elevated in glioma. Furthermore, OS and DFS analyses illustrated that NAP1L1 overexpression was an unfavorable factor associated with reduced survival time of glioma patients. These results indicated that NAP1L1 is an important putative oncogene in glioma. The RT-qPCR analysis also confirmed that the mRNA level of NAP1L1 was dramatically upregulated in 24 gliomas tissues as compared to the corresponding 24 para-tumor tissues. A tissue microarray (TMA) containing 108 glioma and 24 para-tumor tissue samples was used to assess the NAP1L1 expression level. Next, we found that downregulated NAP1L1 attenuated the proliferative ability of glioma cells *in vivo* and *in vitro*. Furthermore, reducing the level of NAP1L1 enhanced the sensitivity to cDDP chemotherapy in glioma cells. Our results indicated that NAP1L1 may be a tumor promoter that could participate in glioma pathogenesis. Prior investigation from our group using mass spectrometry combined with exogenous co-IP, indicated that HDGF was a potential interacting partner of NAP1L1 in endometrial carcinoma (unpublished data). To better understand the underlying molecular mechanisms, we used endogenous co-IP and micro-confocal co-localization assays to confirm NAP1L1 binding with HDGF and their colocalization in the cytoplasm. The findings demonstrated that NAP1L1 interacted with HDGF in glioma.

HDGF is an important oncogene involved in proliferation, invasion, and metastasis in liver cancer, stomach cancer, prostate cancer, and non-small cell lung cancer [20–23]. Our previous study indicated that patients with glioma had a poor prognosis when expression of HDGF was abnormal [24]. These results demonstrated the significance of HDGF in gliomas pathogenesis. In this study, we verified that the mRNA levels of HDGF were increased in 24 gliomas as compared to the corresponding 24 para-tumor tissue samples (Supplementary Figure 2G). Consistent with the mRNA levels, expression of the HDGF protein was dramatically upregulated in glioma. We also found that the expression of HDGF was positively correlated with the tumor WHO grade, as shown by the dramatical differences between high- and low-grade glioma patients. Nonetheless, the specific details of HDGF-induced proliferation of glioma remain unexplored. To examine the HDGF-mediated promotion of glioma cell proliferation, the BIOGRID database was used to predict the interacting protein partners of HDGF, and c-Jun was identified as a potential candidate. Indeed, the interaction of HDGF and c-Jun in glioma cells was confirmed by endogenous co-IP assay. Immunofluorescence analyses illustrated that c-Jun and HDGF proteins mostly co-localized in the cytoplasm of glioma cells; minor nuclear distribution was also observed. In addition, knocking down HDGF significantly decreased the expressions of c-Jun/CCND1/CDK4/CDK6 at the protein level. c-Jun is an oncogenic transcription factor, which can modulate the cell cycle and other tumor pathogeneses mechanisms by transcription or suppression of gene expression [25, 26]. Previous studies show that c-Jun regulates cell proliferation in non-small cell lung cancer by targeting CCND1 [27]. CCND1 is a transcription product of c-Jun [28]; it is a cell cycle promoter inducing cell proliferation in tumors [29]. CDK4/CDK6 is activated by CCND1. Next, western blotting was performed to evaluate the reversal upon c-Jun-overexpression on glioma cell proliferation due to HDGF knockdown. Upregulated CCND1/CDK4/CDK6 expressions were confirmed. Taken together, these results demonstrated that HDGF-mediated c-Jun regulation could promote glioma cell proliferation by inducing CCND1/CDK4/CDK6.

Consistent with their previously described roles in glioma cells, we performed the immunohistochemistry assay to examine NAP1L1 and HDGF expressions in 108 gliomas tissues compared to those in 24 para-tumor tissues. High expression of NAP1L1 was positively correlated with WHO grade, KPS, Ki-67 index, and recurrence, which indicated that NAP1L1 could promote glioma progression. Similarly, high expression of HDGF was positively correlated with WHO grade, KPS, Ki-67 index, and recurrence, which suggested that HDGF was also involved in glioma progression. Patients with high NAP1L1 or HDGF expression levels had significantly shortened OS times as indicated by the Kaplan-Meier analysis. Moreover, NAP1L1 expression was positively correlated with the expression of HDGF in glioma tissues. Glioma patients having high expression of both NAP1L1 and HDGF showed the worst survival prognoses as compared to other groups. These data further confirmed that enhanced NAP1L1 and HDGF expressions were important factors that synergistically promoted the development and spread of glioma.

**Figure 7:**
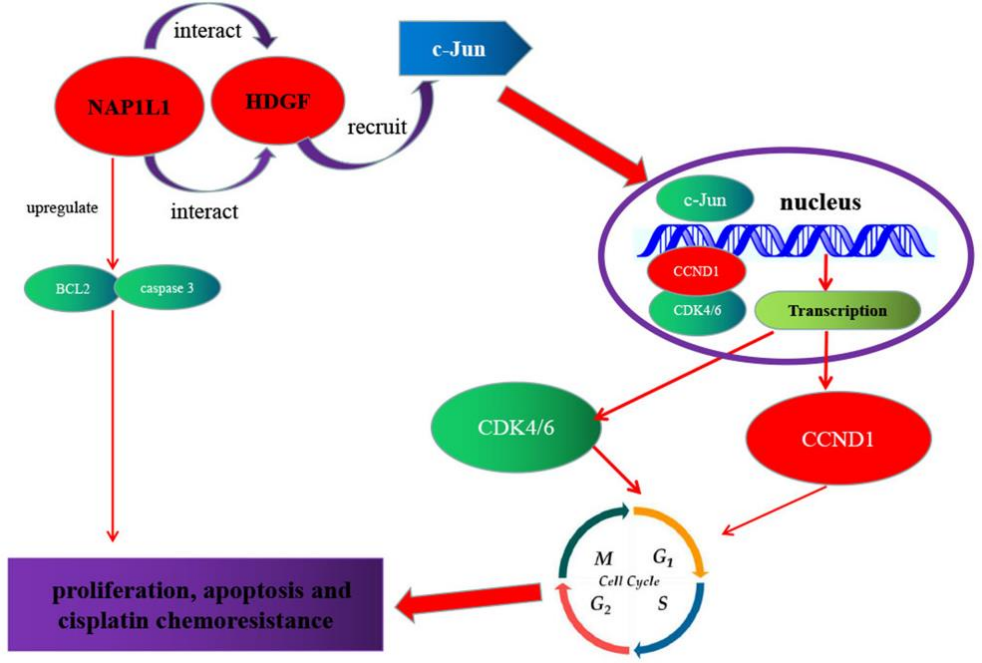
**Schematic of NAP1L1 promoting glioma development.** NAP1L1 interacts with HDGF, while the latter recruits c-Jun, a key oncogenic transcription factor that can induce CCND1/CDK4/CDK6 expression, and thus promotes proliferation and chemoresistance of glioma cells.

As shown in Figure 7, a regulatory model is illustrated. NAP1L1 interacts with HDGF, and the latter recruits c-Jun, a key oncogenic transcription factor, thereby inducing CCND1/CDK4/CDK6 expressions and promoting proliferation and chemoresistance in glioma cells. The high expressions of NAP1L1 or/and HDGF in glioma tissues indicate shorter overall survival in glioma patients.

In summary, NAP1L1 could promote proliferation and chemoresistance in glioma cells by interacting with HDGF and activating c-Jun to induce the expressions of CCND1/CDK4/CDK6. Thus, NAP1L1 has the potential to be a promising biomarker as well as therapeutic target for the pathogenesis of glioma. In the future, more studies are required to further evaluate the functions of NAP1L1.

## MATERIALS AND METHODS

### Cells and patients

Human glioma cell lines U87 and LN229 were obtained from the Cancer Research Institute of Southern Medical University and cultured in DMEM medium containing 10% fetal bovine serum (ExCell, Uruguay) at 37°C with 5% CO_2_. Moreover, glioma and para-tumor tissues were obtained from patients undergoing a surgical procedure at the Nanfang Hospital, Southern Medical University. Ethics approval from the Ethics Committee of the Nanfang Hospital and patient consent were obtained in this study.

### Lentivirus production and infection

Lentiviral vectors harboring shRNA-targeting NAP1L1 and negative control vectors were both established by GeneChem (Shanghai, China). U87 and LN229 cell lines stably expressing shRNA or negative control (NC) vectors were performed as described in a previous study. The NAP1L1 shRNA sequence are presented in Supplementary Table 1. Infection efficiency was showed by green fluorescent protein ratio. The expression of shNAP1L1 was measured by RT-qPCR.

### Transient transfection using plasmids or small interfering RNAs

NAP1L1 and c-Jun plasmids were generated by Guangzhou IGE (IGE Inc., China). Small interfering RNA (siRNA) for NAP1L1, HDGF (named as siNAP and siHDGF, respectively) and the control sequences (siNC) were designed by Guangzhou RiboBio (RiboBio Inc., China). The siNAP1L1, siHDGF sequences are presented in Supplementary Table 2. According to the manufacturer’s protocol, siRNA or plasmids were transfected into U87 and LN229 cells using Lipofectamine^®^ 3000 (Invitrogen; Thermo Fisher Scientific, Inc., Waltham, MA, USA). Cells were collected after 48–72 h for further experiments.

### Reverse transcription-quantitative polymerase chain reaction (RT-qPCR)

Total mRNA was obtained from clinical fresh tissues or cultured cells using QIAZOL (Qiagen, Shanghai, China). And 1 μg extracted mRNA was used for Complementary DNA (cDNA) synthesis with random primers and Maxima First Strand cDNA Synthesis Kit (Takara Bio, Inc., Otsu, Japan). Real-time quantitative PCR (RT-qPCR) was performed according to the manufacturer’s instructions using the SYBR Green Master Mix (TAKARA) and LightCycler480II system (Roche). GAPDH used as the reference gene for NAP1L1 or HDGF or c-Jun; and the relative expression of RNAs was calculated using formula [30]. The primers were listed in the Supplementary Table 3.

### MTT assay

MTT assay was used to perform drug sensitivity tests and cell proliferation. This assay was conducted as described previously [31]. In brief, about 2,000 glioma cells were seeded per well in 96-well plates. After cell adherence, the cells were incubated with MTT at 37°C for 4 h to produce formazan. Then the formazan crystals formed by viable cells were solubilized in 150 μl dimethyl sulfoxide (Sigma). Finally, the absorbance value (OD) was measured at 490 nm by Universal Microplate Reader (Bio-Tek instruments, Inc., Winooski, VT, USA).

### EdU analysis

The Cell-Light EDU Apollo 488 or 567 *in vitro* Imaging kit (Guangzhou Ribobio Co., Ltd.) was used to measure glioma cells proliferation, according to the manufacturer’s introduction. Briefly, following incubation with 10 mM EdU for 2 h at 37°C with 5% CO_2_, the U87 and LN229 cells were fixed with 4% paraformaldehyde, permeabilized with Triton X-100 (0.2%), and stained with Apollo fluorescent dyes and costained with 5 μg/mL DAPI. Finally, EdU-positive cells were counted under a fluorescence microscope in five random fields. All experiments were independently performed at least three times.

### Clone formation assays

U87 and LN229 cells were plated in six-well plates at a density of 1,000 per well, followed by incubation at 37°C in 5% CO_2_ for two weeks. The colonies were fixed with 0.4% gluteraldehyde and stained with 0.1% crystal violet for 15 min. Then the number of colonies containing ≥50 cells was counted under a microscope. This assay was performed as described previously [32].

### Cell apoptosis and cell cycle distribution analysis

Cell apoptosis and cell cycle distribution of the indicated cells were respectively determined using the cell cycle detection kit (BD Biosciences, San Jose, CA) or Annexin V Apoptosis Detection Kit (BD), according to the manufacturer’s instructions by a BD LSRFortessa X-20 (BD). The data were analyzed using FlowJo software version 10.4 (Tree Star, Inc., San Carlos, CA).

### Mitochondrial membrane potential assay

The U87 and LN229 cells were seeded in confocal culture dishes and cultured during the night. In brief, according to the manufacturer’s introduction (Yitabio, Beijing, China), U87 and LN229 cells were stained with JC-10. Then use a Carl Zeiss LSM800 confocal laser scanning microscope to test the samples. The Fiji software is used for the statistical analysis of optical density [33].

### Immunofluorescence and confocal microscopy

The U87 and LN229 cells were separated and seeded on a 35 mm glass bottom cell culture dish (SORFA, ZJ, China) at a density of 4000 cells/well. After cell adherence, the U87 and LN229 cells were fixed with paraformaldehyde (4%) and permeabilized in 0.2% Triton X-100. Then the cells were incubated with specific antibodies (The antibodies are presented in Supplementary Table 4), counter stained with DAPI (0.2 mg/ml), and imaged using a Carl Zeiss LSM800 confocal laser scanning microscope.

### Co-immunoprecipitation (co-IP)

The U87 and LN229 cells cultured in six-well plates were lysed with 600 μl Pierce IP Lysis buffer containing protease and phosphatase inhibitor cocktails. According to the manufacturer’s protocol, the Thermo Fisher Scientific Pierce co-IP kit was used to carry out co-IP. Total proteins were collected from cells and used for protein quantification. The specific anti-HDGF, NAP1L1, c-Jun and normal rabbit IgG (Supplementary Table 4) antibodies (10 μg) was combined with 5 mg protein to incubate overnight. The recovered proteins were analyzed by western blotting as described previously [34].

### Western blotting

Protein was extracted from glioma cells using radioimmunoprecipitation assay buffer (Beyotime Institute of Biotechnology) containing PMSF (Bio-Rad Laboratories, Inc.) and Phosphatase inhibitors (Bio-Rad Laboratories, Inc.) (100:1:1). Then proteins were separated by SDS-PAGE gel electrophoresis and transferred to a PVDF membrane (Beyotime Institute of Biotechnology). Next, the indicated primary antibodies were used for immuno-detection with horseradish peroxidase-conjugated goat anti-rabbit or anti-mouse IgG antibodies and electrochemiluminescence chromogenic kit (Beyotime Institute of Biotechnology). The antibodies used for western blot were showed in the Supplementary Table 4. The experiments were repeated at least three times.

### Animal studies *in vivo*

The 4-week-old male nude mice (BALB/c, male) were purchased from the Experimental Animal Center of Southern Medical University (Guangzhou, China). The animals were maintained under a controlled temperature (20 ± 2°C) with 12-h light/12-h dark cycles and ad libitum access to food and water. To study tumor growth, a subcutaneous xenograft mouse model was established. A total number of 2.0 × 106 U87 or LN229 cells were suspended in 100 μl PBS and subcutaneously injected into the flanks of 4-week-old male nude mice (*N* = 5 per group; left side, sh-NC; right side, shNAP). After 6 days, the tumors were measured using a caliper, and tumor volume was calculated as follows: V = L × W2 × 0.5236, where L is the length and W is the width of the tumor. After 30 days, the tumors were harvested, weighed and photographed. All animal protocols were approved by the Institutional Animal Ethical Committee, Experimental Animal Center of Southern Medical University.

### Immunohistochemical staining (IHC)

Paraffin sections of mouse tumors were used to assess the protein expression of NAP1L1, Ki67 and PCNA by immunohistochemistry assays. According to the manufacturer’s introduction, the indirect streptavidin-peroxidase method was used for immunohistochemistry assays. The assay was completed as previously described [35]. Immunohistochemical staining intensity was evaluated separately by two professional pathologists. A staining score of ≥6 was classified as high NAP1L1 expression and a staining score of ≤4 was considered to have low NAP1L1 expression. The antibodies are presented in Supplementary Table 4.

### Database

The RNA-seq V2 expression data and corresponding clinical information of TCGA CCA cohorts (163 tumor and 207 non-tumor samples) were attained from Gene Expression Profiling Interactive Analysis (GEPIA2) data portal (http://gepia2.cancer-pku.cn/#index). According to the quartile cut point of the expression of NAP1L1, they were divided into 2 groups: weak expression of NAP1L1 group (the first quartile group, *n* = 41) and strong expression of NAP1L1 group (the fourth quartile group, *n* = 41), then the survival analysis was statistically analyzed.

### Statistical analysis

Each experiment was performed with more than three times and data are expressed as the means ± SD. Statistical significant difference was conducted using GraphPad Prism 9.1 (GraphPad Software, Inc.) and IBM SPSS Statistics version 23.0 (IBM SPSS, Inc., Chicago, IL, USA) software. One-way ANOVA or Student’s two-tailed *t* test was performed for comparison between groups. Statistical significance is indicated for each graph (n s, *P* > 0.05; ^*^P < 0.05; ^**^P < 0.01; ^***^P < 0.001), which pointed to its potential role in this type of human malignancy.

### Availability of data and materials

The datasets used and/or analyzed during the current study are available from the corresponding author on reasonable request.

### Ethics approval and consent to participate

The Ethics Committee of The Integrated Hospital of Traditional Chinese Medicine, Southern Medical University authorized the experimental and research protocols of this study. All procedures performed in this study were according with the ethical standards of the institutional research committee and with the 1964 Helsinki declaration and its later amendments or comparable ethical standards. Written informed consent was provided and signed by all patients prior to sample collection. All animal experiments were conducted strictly according with the recommendations in the Guide for the Care and Use of Laboratory Animals of Southern Medical University.

## Supplementary Materials

Supplementary Figures

Supplementary Tables
