# Supplementary material for: NAP1L1 promotes proliferation and chemoresistance in glioma by inducing CCND1/CDK4/CDK6 expression through its interaction with HDGF and activation of c-Jun
Source: Aging (Albany NY). 2021 Dec 27;13(24):26180–200. doi: 10.18632/aging.203805 (PMC8751585; doi:10.18632/aging.203805)
Supplement: Supplementary Figures [file aging-13-203805-s001.pdf]

SUPPLEMENTARY FIGURES

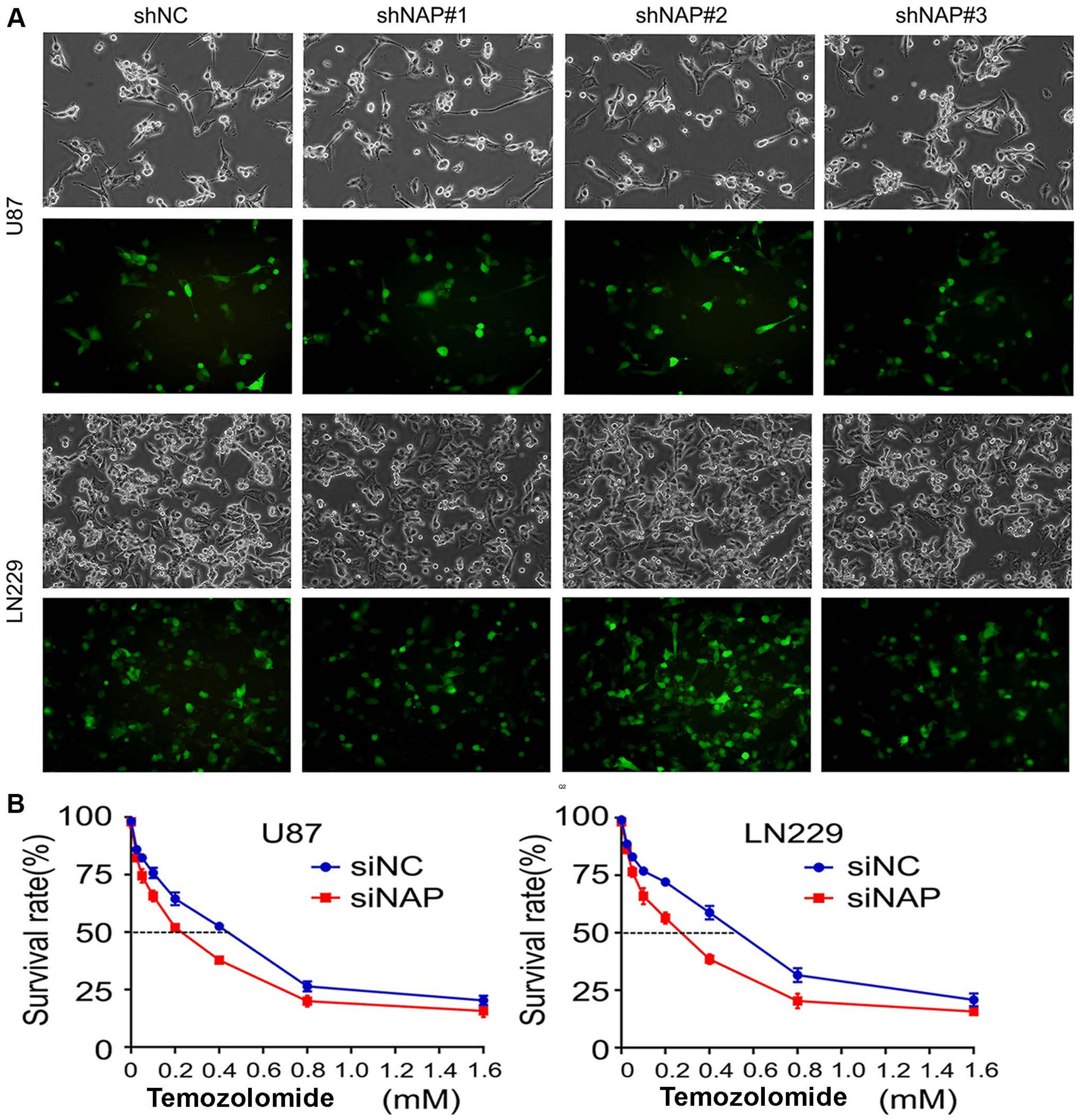

Supplementary Figure 1. (**A**) Representative images of U87 and LN229 cells after transfecting lentiviruses containing shNC or shNAP. Scale bar: 25 μm. (**B**) Dose-response curves of U87 and LN229 treated with shNAP and shNC respectively following treatment with temazolamide for 48 h. Data are presented as the mean ± SD for three independent experiments. $^{*}P < 0.05$, $^{**}P < 0.01$, $^{***}P < 0.001$.

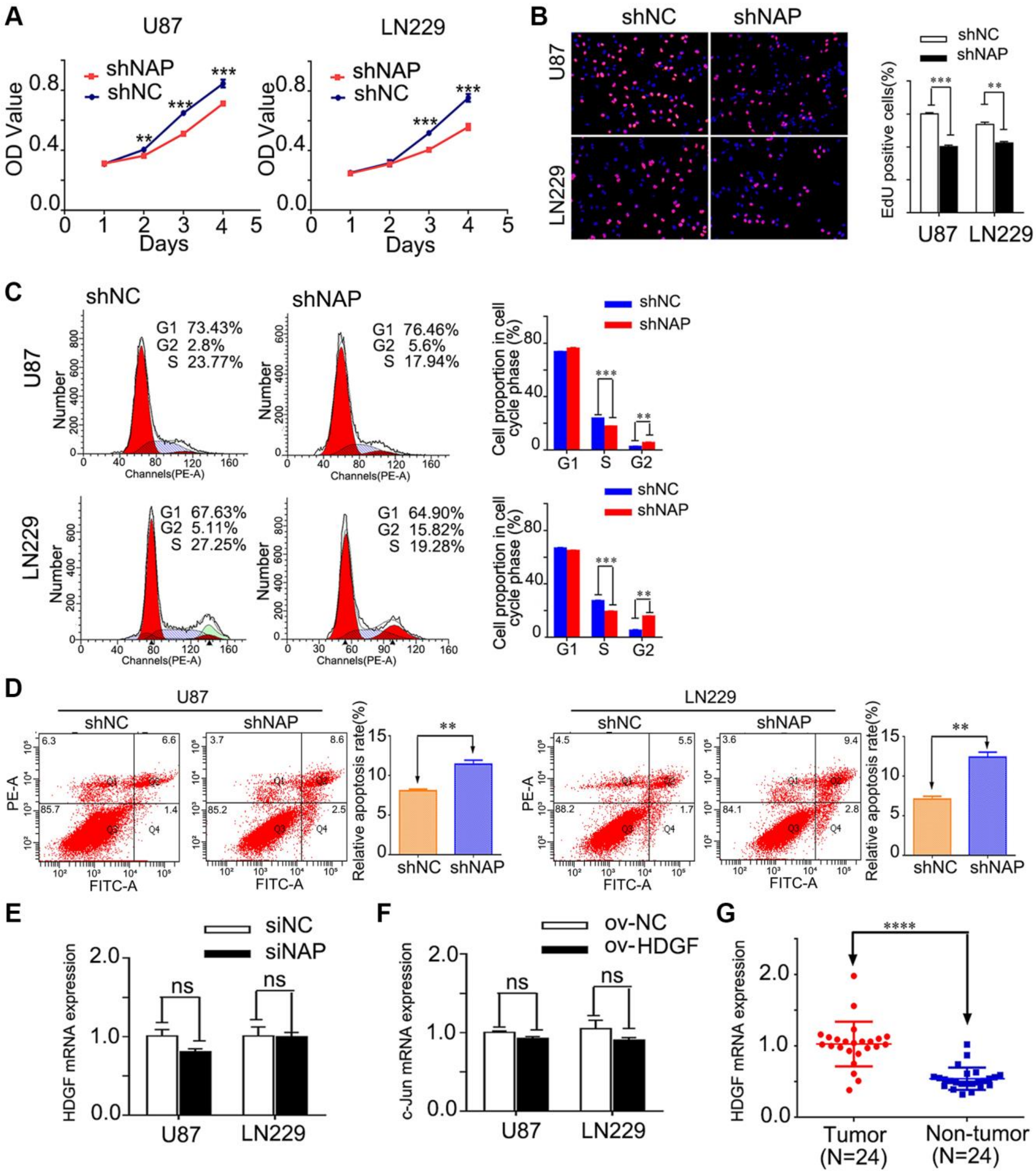

Supplementary Figure 2. Suppressing NAP1L1 inhibits glioma cell proliferation, blocks the cell cycle and induces apoptosis. (**A**) U87 and LN229 cells were transfected with shNC or shNAP lentiviruses, and then subjected to MTT assay. (**B**) The proliferation of U87 and LN229 cells transfected with lentiviruses containing shNC or shNAP is measured using the EdU assays. (**C**, **D**) The cell cycle distribution (**C**) and apoptotic fraction (**D**) in U87 and LN229 cells transfected with lentiviruses containing shNC or shNAP is analyzed by flow cytometry. (**E**) RT-qPCR analysis of HDGF mRNA expression in U87 and LN229 cells transfected with siNAP1L1. (**F**) RT-qPCR analysis of c-Jun mRNA level in U87 and LN229 cells transfected with HDGF-overexpressing plasmid. (**G**) RT-qPCR analysis of HDGF mRNA expression in 24 glioma tissues and 24 para-tumor tissues. Data are presented as the mean ± SD for three independent experiments. $^{*}P < 0.05$, $^{**}P < 0.01$, $^{***}P < 0.001$.
