# Supplementary material for: NAP1L1 promotes proliferation and chemoresistance in glioma by inducing CCND1/CDK4/CDK6 expression through its interaction with HDGF and activation of c-Jun
Source: Aging (Albany NY). 2021 Dec 27;13(24):26180–200. doi: 10.18632/aging.203805 (PMC8751585; doi:10.18632/aging.203805)
Supplement: Supplementary Tables [file aging-13-203805-s002.pdf]

SUPPLEMENTARY TABLES

Supplementary Table 1. shRNA sequences of NAP1L1.

NO.	5′	STEM	Loop	STEM	3′
NAP1L1-RNAi(78814)-a	Ccgg	gcCAAGATTGAAGA TGAGAAA	CTCGAG	TTTCTCATCTTCA ATCTTGGC	TTTTTg
NAP1L1-RNAi(78814)-b	aattcaaaaa	gcCAAGATTGAAGA TGAGAAA	CTCGAG	TTTCTCATCTTCA ATCTTGGC	
NAP1L1-RNAi(78815)-a	Ccgg	TTGATAAGCGATTT GAAATTA	CTCGAG	TAATTTCAAATCG CTTATCAA	TTTTTg
NAP1L1-RNAi(78815)-b	aattcaaaaa	TTGATAAGCGATTT GAAATTA	CTCGAG	TAATTTCAAATCG CTTATCAA	
NAP1L1-RNAi(78816)-a	Ccgg	TTCCAATGACTCTT TCTTTAA	CTCGAG	TTAAAGAAAGAG TCATTGGAA	TTTTTg
NAP1L1-RNAi(78816)-b	aattcaaaaa	TTCCAATGACTCTT TCTTTAA	CTCGAG	TTAAAGAAAGAG TCATTGGAA	

Supplementary Table 2. siRNA sequences of NAP1L1 and HDGF.

Gene	NO	Target Sequence
NAP1L1	genOFFTM st-h-NAP1L1_001	GAAGTATGCTGTTCTCTAT
	genOFFTM st-h-NAP1L1_002	CCAACAGGATACATTGAAA
	genOFFTM st-h-NAP1L1_003	GACAGTTCGTACTGTGACT
HDGF	HDGF-RNAi-1	5′GAAACGAGAUCGAAUGCAC dTdT 3′
	HDGF-RNAi-2	5′CUCAAGCGUUUCCUCCUUA dTdT 3′
	HDGF-RNAi-3	5′CCAUACGAUUGACGAGAUG dTdT 3′

Supplementary Table 3. The primers used in this study.

Primers name		Sequence (5′–3′)
NAP1L1	Forward	TTTGCCCCTCCTGAAGTTCC
	Reverse	CCCAACACAACTTGAGACATCC
HDGF	Forward	ATCAACAGCCAACAAATACC
	Reverse	TTCTTATCACCGTCACCCT
c-JUN	Forward	TCAGACAGTGCCCGAGATG
	Reverse	CTGCTGCGTTAGCATGAGTT
GAPDH	Forward	CATGGGTGTGAACCATGAGA
	Reverse	GTCTTCTGGGTGGCAGTGAT

Supplementary Table 4. A list of antibodies used for WB, IF, CoIP and IHC.

Antibody	Cat. No	Company	Species	Dilution
NAP1L1	mAb ab 178687	Abcam	Rabbit	1:1000(WB); 1:200(IF); 1:20(CoIP)
NAP1L1	ab 33076	Abcam	Rabbit	1:1000(WB); 1:100(IF); 1:300(IHC)
NAP1L1	pAb #14898-1-AP	Proteintech	Rabbit	1:1000(WB); 1:100(IF); 1:300(IHC)
HDGF	mAb #60064-1-Ig	Proteintech	Mouse	1:1000(WB); 1:50(IF); 1:10(CoIP)
HDGF	pAb 11344-1-AP	Proteintech	Rabbit	1:1000(WB); 1:200(IHC)
c-Jun	mAb #9165	Cell Sigaling	Rabbit	1:1000(WB); 1:50(IF); 1:10(CoIP)
c-Jun	24909-1-AP	Proteintech	Rabbit	1:1000(WB); 1:100(IF); 1:10(CoIP)
c-Jun	28891-1-AP	Proteintech	Mouse	1:1000(WB); 1:100(IF); 1:10(CoIP)
CCND1	mAb #6086-1-Ig	Proteintech	Mouse	1:1000(WB)
PCNA	mAb #13110	Cell Sigaling	Rabbit	1:300(IHC)
GAPDH	pAb AP0063	Bioworld	Rabbit	1:1000(WB)
BCL2	ab182858	Abcam	Rabbit	1:1000(WB)
Ki-67	Ab16667	Abcam	Mouse	1:200(IHC)
CDK4	ab199728	Abcam	Rabbit	1:1000(WB)
CDK6	ab241554	Abcam	Mouse	1:1000(WB)
Cleaved Caspase3	Ab214430	Abcam	Rabbit	1:1000(WB)
